# Zn doped iron oxide nanoparticles with high magnetization and photothermal efficiency for cancer treatment†

**DOI:** 10.1039/d2tb01338j

**Published:** 2022-10-12

**Authors:** Georgios Kasparis, Anouchka Plan Sangnier, Lilin Wang, Christoforos Efstathiou, Alec P. LaGrow, Andreas Sergides, Claire Wilhelm, Nguyen Thi Kim Thanh

**Affiliations:** a Biophysics Group, Department of Physics and Astronomy, University College London Gower street London WC1E 6BT UK ntk.thanh@ucl.ac.uk; b UCL Healthcare Biomagnetic and Nanomaterials Laboratories 21 Albemarle street London W1S 4BS UK; c Laboratoire Physico Chimie Curie, PCC, CNRS UMR168, Institut Curie, Sorbonne University, PSL University 75005 Paris France; d Inserm, U1148, Laboratory for Vascular Translational Science, Université Paris 13, Sorbonne Paris Cité Bobigny F-93017 France claire.wilhelm@curie.fr

## Abstract

Magnetic nanoparticles (NPs) are powerful agents to induce hyperthermia in tumours upon the application of an alternating magnetic field or an infrared laser. Dopants have been investigated to alter different properties of materials. Herein, the effect of zinc doping into iron oxide NPs on their magnetic properties and structural characteristics has been investigated in-depth. A high temperature reaction with autogenous pressure was used to prepare iron oxide and zinc ferrite NPs of same size and morphology for direct comparison. Pressure was key in obtaining high quality nanocrystals with reduced lattice strain (27% less) and enhanced magnetic properties. Zn_0.4_Fe_2.6_O_4_ NPs with small size of 10.2 ± 2.5 nm and very high saturation magnetisation of 142 ± 9 emu g_Fe+Zn_^−1^ were obtained. Aqueous dispersion of the NPs showed long term magnetic (up to 24 months) and colloidal stability (at least 6 d) at physiologically mimicking conditions. The samples had been kept in the fridge and had been stable for four years. The biocompatibility of Zn_0.4_Fe_2.6_O_4_ NPs was next evaluated by metabolic activity, membrane integrity and clonogenic assays, which show an equivalence to that of iron oxide NPs. Zinc doping decreased the bandgap of the material by 22% making it a more efficient photothermal agent than iron oxide-based ones. Semiconductor photo-hyperthermia was shown to outperform magneto-hyperthermia in cancer cells, reaching the same temperature 17 times faster whilst using 20 times less material (20 mg_Fe+Zn_ ml^−1^*vs*. 1 mg_Fe+Zn_ ml^−1^). Magnetothermal conversion was minimally hindered in the cellular confinement whilst photothermal efficiency remained unchanged. Photothermia treatment alone achieved 100% cell death after 10 min of treatment compared to only 30% cell death achieved with magnetothermia at clinically relevant settings for each at their best performing concentration. Altogether, these results suggest that the biocompatible and superparamagnetic zinc ferrite NPs could be a next biomaterial of choice for photo-hyperthermia, which could outperform current iron oxide NPs for magnetic hyperthermia.

## Abstract 2

The substitution of Fe^2+^ ions by Zn^2+^ ions in the crystal lattice of ferrites increases both the nanoparticles magnetization, and their photothermal conversion efficiency.

## Graphical abstract

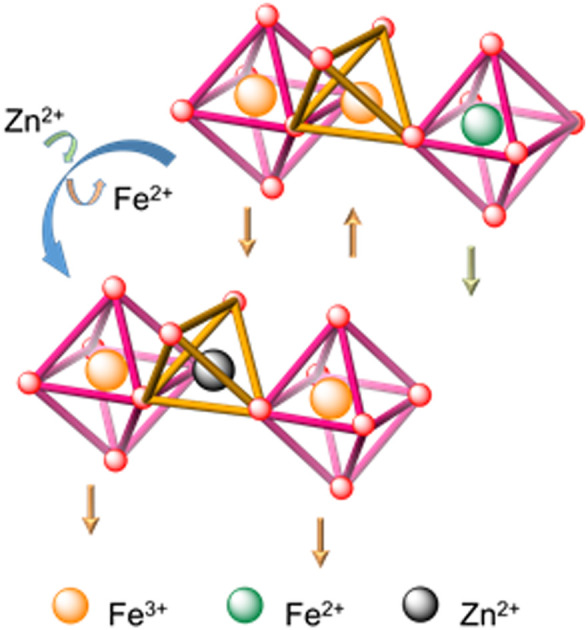

## Introduction

Magnetic NPs are of interest in various fields and their saturation magnetization (*M*_S_) is of paramount importance in many applications such as drug delivery, bio-separation, bio-engineering, imaging, and magnetic fluid hyperthermia (MFH).^1–8^ More complex systems are pursued to enhance their magnetic properties including metallic core NPs,^9^ their alloys,^10^ core–shell architectures,^11^ or encapsulation.^12,13^ Some of these complex nanomaterials might experience some toxicity *in vivo*,^14^ so that, to date, only iron oxide NPs have been approved by the Food and Drug Administration (FDA) and European Medicines Agency (EMA) despite them having limited magnetic properties. Alternative materials to either iron oxide or metallic core NPs are doped ferrites; different metal cations are introduced into the lattices to alter their properties. Such materials take the general form M_*x*_Fe_3−*x*_O_4_ where M is the dopant cation such as Ni^2+^, Mn^2+^, Zn^2+^, Co^2+^, Mg^2+^, and Cu^2+^.^15^ Doping can change the structural,^16^ electronic^17^ and magnetic properties of materials.^18,19^ Although many of them might find use in solar cells^17^ or batteries,^16^ dopants for biomedical applications are limited due to the inherent toxicity of some elements.^14,20,21^

Larger NPs in the superparamagnetic limit are pursued to achieve a smaller spin-canted layer-to-volume ratio which minimizes the reduction in the observed saturation magnetization (*M*_S_) value due to canting effect.^22^ Smaller sized NPs were shown to have prolonged systemic circulation,^23–27^ ability to cross the blood-brain barrier,^28^ enhanced cellular uptake and sub-cellular size-dependent localization compared to their larger analogues.^29^ There is a challenging need for a facile, reproducible and scalable synthesis for the production of highly magnetic and biomedically relevant NPs.

Zinc has the second highest abundance in the human body after iron among the d-block elements and its deficiency poses several risks to health such as oxidative stress and DNA damage.^30,31^ Its importance in many enzymatic processes and food fortification with Zn^2+^ indicate the high tolerance to and a need of Zn^2+^ for the human body to function properly.^32^ Therefore, Zn^2+^ would be a biocompatible dopant for magnetic ferrite NPs. Herein, we demonstrate a high temperature reaction with autogenous pressure for the preparation of small, highly crystalline and biocompatible zinc ferrite NPs. We report the preparation of zinc ferrite NPs with ten times less magnetic volume (volume of magnetic material) but have similar *M*_S_ as the current highest reported value for the same material.^33^ The high reproducibility of the reaction and the ability to prepare iron oxide and zinc ferrite NPs with the same size and morphology allowed for an in-depth study of their structural and magnetic properties such as unit cell parameter, lattice strain, saturation magnetization (*M*_S_), Curie temperature (*T*_c_), coercivity (*H*_c_), blocking temperature (*T*_B_) and effective, surface and magnetocrystalline anisotropies. The surface of these high quality NPs was functionalized with citrate in a one pot functionalization step producing NP dispersions whose magnetic and colloidal stability were assessed. The produced NPs show no signs of cytotoxicity up to 200 μg_Fe+Zn_ ml^−1^ and a viability of 80% at 1500 μg_Fe+Zn_ ml^−1^ based on metabolic activity. Similar results were obtained when assessing membrane integrity, while the clonogenicity of cells is not affected by the NPs at 200 μg ml_Fe+Zn_^−1^. These biocompatible-proven NPs show augmented performance compared to currently investigated iron oxide NPs in photohyperthermia (PT), a recent alternative cancer thermotherapy using magnetic NPs achieving total cell death in a short time (10 min). Moreover, PT was compared in-depth with the more typical MFH modality and showed an impressive, enhanced performance.

## Experimental

### Materials

Fe(acac)_3_ (99.0%), Zn(acac)_2_·*x*H_2_O (99.995% trace metal basis), 70% HNO_3_ for inductively coupled plasma (ICP) spectroscopy (99.999% trace metal basis) and 1,10-phenanthroline monohydrate (99%), triethylene glycol (TREG) (ReagentPlus® 99%), Zn standard for atomic emission spectroscopy (OES) (TraceCERT®), Fe standard for ICP (TraceCERT®), hydroxylamine hydrochloride (ReagentPlus® 99%), nuclear fast red solution (0.1% in 5% aluminium sulfate), potassium hexacyanoferrate(ii) trihydrate (98.5% ACS reagent), crystal violet solution for staining, sodium cacodylate trihydrate (98%), trypan blue 0.4% solution and sucrose (99.5%) were purchased from Merck (UK). 37% HCl was purchased from Acros Organics (UK). Acetone (technical grade) was purchased from VWR (UK). Trisodium citrate (99%) was purchased from BDH (UK). FeCl_2_·4H_2_O (99.0%) was purchased from Honeywell (UK). Water was purified by Purelab Ultra, Elga. Eagle minimum essential medium (EMEM), dimethyl sulfoxide (DMSO) (BioReagent, 99.9%) and sodium acetate anhydrous (ReagentPlus® 99.0%) were purchased from Sigma Life Science (UK). Fetal bovine serum (FBS), penicillin/streptomycin (P/S), GlutaMAX, Trypsin-EDTA 0.05%, phosphate-buffered saline (PBS) and Dulbecco's Modified Eagle Medium (DMEM) were purchased from Gibco (UK). 3-(4,5-Dimethylthiazol-2-yl)-2,5-diphenyltetrazolium bromide (MTT) (98%) was purchased from Alfa Aesar (UK). Paraformaldehyde (PFA) (96.5%) was purchased from TAAB (UK). Alamar Blue was purchased from Invitrogen. Pierce LDH cytotoxicity assay kit was purchased from Thermo Scientific (UK). Fluorsave antifade solution was purchased from Calbiochem (UK). All chemicals have been used without further treatment.

### NP synthesis

For the synthesis of Zn_0.4_Fe_2.6_O_4_ NPs, Fe(acac)_3_ (1.23 g, 3.5 mmol) and Zn(acac)_2_·*x*H_2_O (0.14 g, 0.5 mmol) were dispersed in TREG (20 ml) by inversion and short bath sonication. The mixture was transferred into a 45 ml Teflon liner and assembled with an autoclave jacket. The mixture was heated to 250 °C at a rate of 5 °C min^−1^ using a Memmert UFP400 oven and kept at that temperature for 8 h after which it was let cool to room temperature. The NPs were precipitated with acetone using a 1 : 8 reaction mixture : acetone volume ratio at a centrifugal force of 9000 g for 10 min three times or with magnetic decantation.

The synthesis of NPs under ambient pressure used the same reaction mixture in a three-necked flask equipped with a Liebig condenser. Under no stirring the flask was heated to the same temperature, rate and duration controlled by a Julabo LC6 PID controller equipped with two platinum probes.

### Yield quantification

Powder samples underwent elemental analysis with ICP-optical emission spectroscopy (OES) to determine the metal fraction in the samples. The theoretical metal content was used as 100% conversion of the metallic content of the precursor to nanoparticulate form. The percent yield was calculated according to eqn (1).1
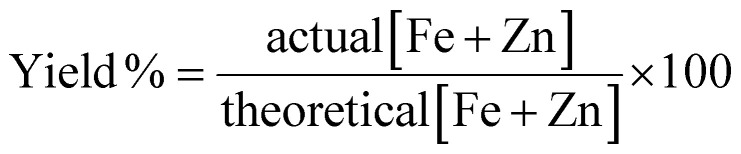


### Surface functionalisation

The crude NP suspension could be functionalized by thermally equilibrating 8 ml of the suspension at 70 °C with intense magnetic stirring at 1300 rpm. An equal volume of trisodium citrate (0.2 M) solution was added and the mixture was stirred for further 2 h. The product was collected by magnetic decantation and washed with acetone (2 × 10 ml) after which the NPs were dispersed in a minimum volume of water to form stable ferrofluids (Fig. S1B, ESI†).

### Elemental analysis

The metallic content was assessed by ICP-OES and UV-Vis spectroscopy. For ICP-OES, a solid or liquid sample was digested in 70% HNO_3_ and diluted to 1% for measurements. A calibration plot was prepared with ICP standards for both iron and zinc metals and measurements were obtained with an Agilent Varian-720-ES ICP-OES spectrometer. For UV-Vis analysis the sample was dissolved in 4 M HCl with mild heating at 65 °C and diluted if necessary. Hydroxylamine hydrochloride solution (50 μl, 0.15 M) were added to sodium acetate (450 μl, 1.5 M) solution. 200 μl of the digested sample were added to the above solution and vortexed to facilitate the reduction of Fe^3+^ to Fe^2+^. Finally, an acidic solution of 1,10-phenanthroline (300 μl, 0.06 M in 0.04 M HCl) was added and the vials were vortexed and stored overnight in the dark to facilitate the complexation. The same procedure was followed to prepare a calibration plot from FeCl_2_·4H_2_O dissolved in 4 M HCl between 0.000625–0.02 mg_Fe_ ml^−1^. Aliquots of 100 μl of each sample were plated in a clear 96-well plate and read at 510 nm on a Molecular devices SpectraMax Me^2^ UV-Vis spectrometer.

### Magnetic properties measurements

Magnetic measurements were performed on a Quantum Design MPMS3 SQUID-VSM. Powder samples were placed in gelatin capsules and immobilized with cotton wool. The gelatin capsule was placed in the interior of a diamagnetic plastic straw and measurements were taken between ±70 kOe. Data were corrected for diamagnetic contribution. Zero field cooled-field cooled (ZFC-FC) measurements were recorded between 5–300 K at an applied field of 50 Oe. The T_C_ was extracted from magnetoTGA (thermal gravimetric analysis) measurements using a Discovery TGA, TA Instruments. Powder samples were placed in a single use aluminium crucible suspended on a platinum pan. The heating rate was set at 10 °C min^−1^ with a chamber nitrogen flow of 10 ml min^−1^ and a sample nitrogen flow of 25 ml min^−1^. The temperature was increased from room temperature to 500 °C to burn off ligands on the surface and then to reveal the *T*_C_ as a sudden decrease in weight due to loss of attraction to the electromagnet operating at 80% of its capacity.

### Electron microscopy

Samples were casted on a carbon-coated copper grid and air dried. The NPs were visualized under a JEOL JEM 1200-EX electron microscope at an accelerating voltage of 120 kV. HRTEM and high angle annular dark field imaging (HAADF) scanning transmission electron microscopy (STEM) with EDX mapping were recorded on a Titan *G*^2^ 60–300 equipped with a coefficient spherical aberration image corrector operating at 300 kV. Elemental mapping was carried out using STEM with a condenser aperture of 70 μm and a windowless silicon drift detector. The sample was tilted 15° towards the EDX detector for acquisition.

### X-Ray diffraction

Diffraction patterns of powder samples were recorded at room temperature on a PANalytical X’Pert^3^ equipped with a cobalt source (*λ* = 1.789 Å) between 20–110° on transmission-reflection spinning mode. The results were fitted and analysed by X’Pert HighScore Plus software. The crystallite size was calculated by the Scherrer equation shown in eqn (2).2
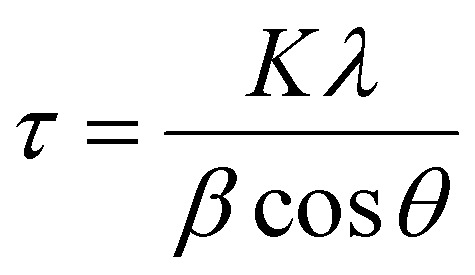
where *τ* is the crystallite size, *K* is the shape factor with a typical value of 0.9, *λ* is the wavelength of the X-ray source, *β* is the line broadening at half the maximum intensity and *θ* is the Bragg angle.

The lattice strain of the NPs was calculated based on the Williamson–Hall analysis as shown in eqn (3).3
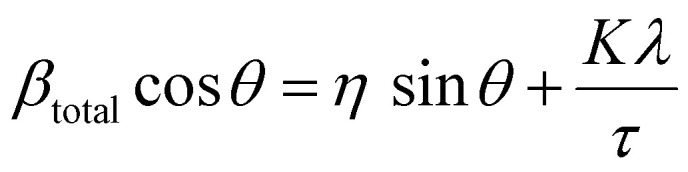
where *β*_total_ is the line broadening arising from all effects, *θ* is the Bragg angle, *η* is the lattice strain, *K* is the shape factor with a typical value of 0.9, *λ* is the wavelength of the X-ray source and *τ* is the crystallite size.

To calculate the lattice parameter, Bragg's law, eqn (4), was used to calculate the interplanar distance, which then relates to the lattice parameter according to eqn (5).

4 *nλ* = 2*d* sin *θ*

5
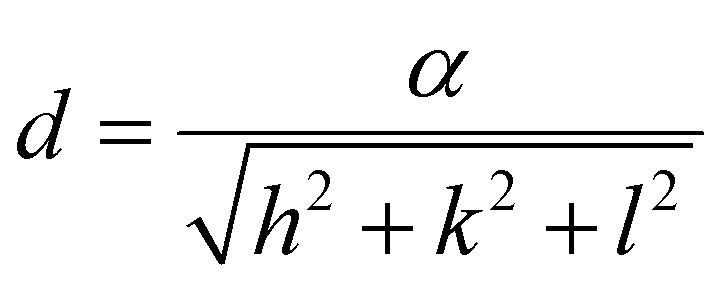
where *n* is a positive integer, *λ* is the wavelength of the X-ray source, d is the interplanar spacing, *θ* is the Bragg angle, *α* is the lattice parameter and *h*, *k*, *l* are Miller indices.

The percentage of Fe_3_O_4_ and γ-Fe_2_O_3_ component in the synthesized Fe_3_O_4_@γ-Fe_2_O_3_ NPs was estimated using Vegard's law shown in eqn (6).6
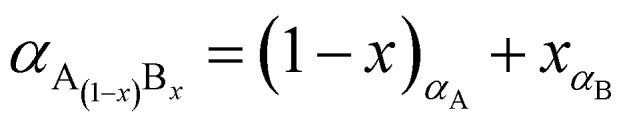
where *α* is the lattice parameter, *x* is the molar fraction and A and B are the two different materials.

### Surface characterisation by infrared spectroscopy and TGA

Surface ligands were identified by PerkinElmer Spectrum 100 attenuated total reflectance Fourier transformed infrared spectrometer (ATR-FTIR) on dry powder samples at ambient conditions. Scans were recorded between 600–4000 cm^−1^ and 20 scans were accumulated for each measurement. Spectra were analysed using the software Spectrum. Quantification of surface ligands was done by TGA under nitrogen up to 500 °C. Data were analysed by Trios software.

### Stability studies by DLS

The hydrodynamic diameter of the functionalized NPs was assessed by dynamic light scattering (DLS) measurements on a Malvern Zetasizer nano ZS operating with a 633 nm He–Ne laser. Solutions with desired pH or electrolyte concentration were prepared and 1 drop of ferrofluid with a concentration of 10 mg_Fe+Zn_ ml^−1^ was added and mixed. The mixture was immediately filtered by syringe filtration (0.2 μm) and the filtrate was monitored for one week. All measurements were recorded at 25 °C in independent triplicates.

### NP uptake

The uptake of NPs was quantified by single cell magnetophoresis. U87-MG and MCF-7 cells were grown to near confluency in T25 flasks in DMEM supplemented with 10% fetal bovine serum, 1% penicillin-streptomycin and 2 mM glutamine (further named complete DMEM). The medium was removed and NPs at a concentration of 0.2 mg_Fe+Zn_ ml^−1^ in complete DMEM medium were added in each flask and incubated between 1–24 h. After, the cells were washed with DMEM (2 × 3 ml) and PBS (2 × 3 ml), and then detached by trypsin and collected by centrifugation (1500 rpm, 3 min). 1 ml of complete DMEM was used to resuspend the cells to singe cell suspension by pipetting and transferred in a 1 mm thick Hellma chamber and subjected to a magnetic field (*B* = 321 mT, gradB = 4.7 T m^−1^, Fig. S2, ESI†) generated by a neodymium permanent bar magnet. The cells are attracted to the magnet by the magnetic force *F* = MgradB, *M* being the magnetic moment. The magnetic force is countered by Stoke drag force and equilibrium is quickly reached, *F*_drag_ = 6π*ηrV*, where *r* is the cell radius, *η* the dynamic viscosity of water and *V* the speed of the cell. Videos of the cell movements were recorded on a Leica DMIL inverted microscope equipped with an MC170 HD camera at 4 images per second. Using the image analysis software ImageJ (NIH) the diameter and distance travelled by cells were measured and by using a material density of 5 × 10^6^ g m^−3^ the amount of iron in each cell could be calculated by converting the cell magnetic moment using the magnetic moment of Zn_0.4_Fe_2.6_O_4_ NPs at the field strength applied by the bar magnet from SQUID-VSM measurements.

### Histology and localization studies

U87-MG cells were grown on glass cover slips in a clear 24-well plate at a seeding density of 1 × 10^5^ cells per well. After overnight incubation, the cells were treated with different concentrations of NPs (0–300 μg_Fe+Zn_ ml^−1^) in complete EMEM for 24 h. After, the cells were washed with medium (2 × 3 ml) and PBS (2 × 3 ml). Fresh complete medium was added to the cells and incubated for further 2–3 h to allow the cells to relax from handling stress. Sucrose supplemented (2%) PFA solution in PBS (100 μl, 4%) was added to each well as a prefixation step and incubated at 37 °C for 10 min. After, the cells were washed with PBS (2 × 3 ml) and the cells were fixed with 100 μl of sucrose-PFA solution. After 10 min incubation at 37 °C the cells were stained with a 1 : 1 mixture of 4% HCl and 4% potassium hexacyanoferrate(ii) trihydrate for 3 min. The cells were washed with water (2 × 100 μl) and counterstained with Nuclear Fast red solution for 5 min. Stained cells were dehydrated with ascending alcohol by immersion in 70% (2 min) and 100% (5 min) ethanol. The cover slips were mounted on microscope slides over anti-fade oil and sealed with nail polish. Images were obtained on an inverted microscope Leica DMI6000B in bright field mode.

### Toxicology assays

The toxicity of the NPs was assessed by MTT, LDH and clonogenic assays. For the MTT assay, U87-MG cells were plated in 96-well plates at a density of 5000 cells per well and incubated for 24 h. The experimental groups were exposed to NP suspensions with concentrations up to 1500 μg_Fe+Zn_ ml^−1^ in complete EMEM with no phenol red and the cells were incubated for further 24 h. Dilution of the culture medium from NP suspension was capped at 10% to prevent viability decrease from osmotic imbalance. The NPs were removed and syringe-filtered (0.2 μm) MTT solution (10 μl, 0.2 mg ml^−1^) was added in each well and incubated for 4 h at 37 °C. After, the MTT solution was removed and DMSO (200 μl) was added to dissolve the purple formazan crystals on a plate shaker for 30 min. 150 μl of the supernatant were transferred in another 96-well plate and the absorbance was measured at 570 nm. The assay was performed in quadruplicate and statistical analysis was performed by two-way Anova using SPSS software.

The LDH assay requires an optimum cell number which needs to fall within the linear part of the absorbance curve which needs to be determined for different cell lines as they have different LDH activity. In conjunction with the cell growth curve and LDH activity with respect to cell number (Fig. S3, ESI†), 3000 cells per well was the optimum seeding number. Cells were seeded in 96-well plates incubated overnight. NPs were added to final concentrations between 0–1000 μg_Fe+Zn_ ml^−1^. EMEM, spontaneous (ultrapure water) and maximum (10× lysis buffer) LDH activity (SA and MA respectively) served as controls. 24 h after, 50 μl of all samples were transferred into a clean 96-well plate and mixed with reaction mixture (50 μl). Wells treated with NPs rest on a bar magnet for 5 min to prevent transferring any NPs to the clean plate. The plate was left at room temperature for 30 min protected from light. Stop solution (50 μl) was added in all wells and the absorbance was measured at 490 nm and 680 nm. The absorbance values at 680 nm were subtracted from the 490 nm values before calculating cytotoxicity as shown in eqn (7).7
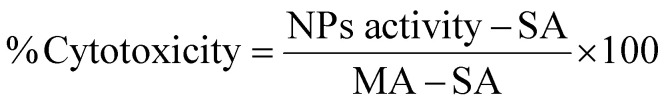


The clonogenic assay was performed by seeding 100 NP-treated U87-MG cells (0.2 mg_Fe+Zn_ ml^−1^, 24 h) as single cell suspension per well of a 6-well plate. Untreated cells were used as controls. The cells were incubated at 37 °C, 5% CO_2_. When large enough colonies were formed (50 cells or more to score), the medium was removed, cells were washed with PBS and fixed with 6% glutaraldehyde for 10 min. Colonies were stained with crystal violet solution for 30 min and washed by immersion in water until clear to the naked eye, let air-dry and counted under a microscope.

### Hyperthermia measurements

Temperature was measured using an infrared thermal imaging camera (FLIR SC7000) in real time every second and processed with the software Altair. Magnetic hyperthermia was performed on a commercially available instrument from nB nanoScale Biomagnetics, Spain. Field and frequencies for the alternating magnetic field were electronically controlled using the software Magno. Photothermia was performed using lasers from Laser Components SAS, France, calibrated to irradiate samples with 0.3 W cm^−2^ and 1 W cm^−2^. Small volumes of samples (50–100 μl) were placed in Eppendorf tubes and treated with either method. The heating power was measured by the temperature plateau (Δ*T*) and specific loss parameter (SLP). The temperature plateau was directly measured by the infrared camera while the SLP value was calculated according to eqn (8).8
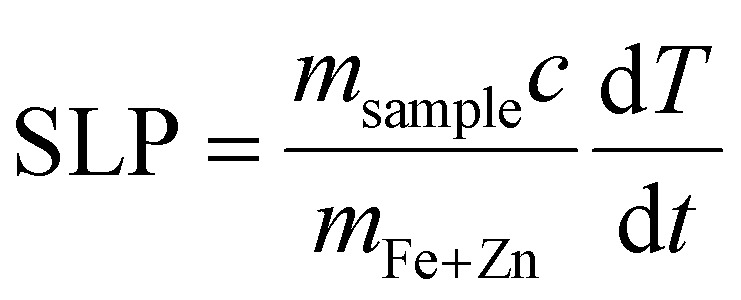
where *m*_sample_ is the total mass of the sample (g), *c* is the specific heat capacity approximated to that of water (4.185 J g^−1^ K^−1^), *m*_Fe+Zn_ is the total mass of iron and zinc in the sample (g) and d*T*/d*t* is the initial slope of the heating curves over the first 30 s. Alternatively, the light to heat coefficient can be calculated according to:9
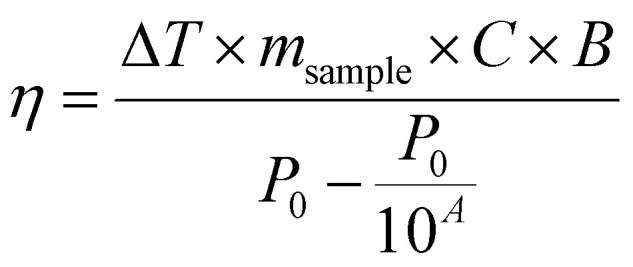
 with *A* the sample absorbance and *B* the constant rate of heat dissipation from the solution to the external environment. *B* was calculated from experiments according to 
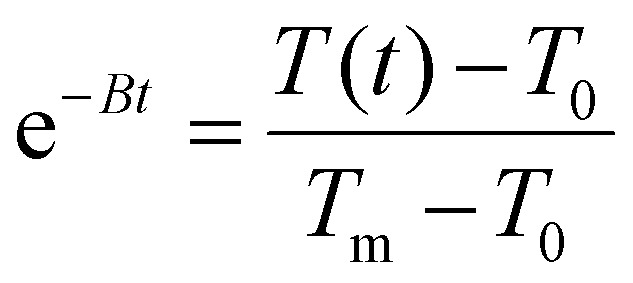
, *T*(*t*) being the temperature at time *t*, *T*_0_ the initial temperature of the sample before photothermal heating and *T*_m_ the maximum temperature reached after 5 min exposure to laser.

The impact of cellular confinement was assessed in cacodylate fixed U87-MG cells. Briefly, cells were grown to near confluency treated with 0.2 mg_Fe+Zn_ ml^−1^ Zn_0.4_Fe_2.6_O_4_ NPs for 24 h, washed with PBS and trypsinised. Fixation was done with 0.2 M sodium cacodylate : 25% glutaraldehyde : water mixture with a volume ratio of 5 : 2 : 3. The cell pellet was resuspended in PBS and treated. For live-cell experiments, cells were treated with NPs, washed with PBS and trypsinised. The cell pellet was treated with MFH or PT (pre-equilibrated at 37 °C), resuspended in DMEM and plated in a 96-well plate. After 24 h, cells were washed with DMEM with no phenol red and incubated with DMEM with no phenol red supplemented with 10% Alamar Blue for 1 h. Then, 50 μl from each well were transferred to a clean plate and fluorescence was measured on a multimode plate reader EnSpire PerkinElmer at an excitation wavelength of 570 nm and fluorescence detection at 585 nm. Alternatively, the cell pellet was incubated with live/dead reagent from live/dead cell imaging kit (ThermoFisher, R37601) according to manufacturer's instruction, and fluorescent image of live (green) and dead (red) cells were acquired. The number of dead cells was counted and expressed as percent of total number of live and dead cells.

## Results and discussion

### NP synthesis under high temperature with autogenous pressure

The synthesis of zinc ferrite NPs under high temperature and autogenous pressure was inspired by a previously developed polyol synthesis in our laboratory producing iron oxide NPs of high *M*_S_ values.^34^ The reaction for the preparation of zinc ferrites involves the thermal decomposition of iron(iii) acetylacetonate, Fe(acac)_3_, and zinc acetylacetonate hydrate, Zn(acac)_2_·*x*H_2_O, in TREG at different ratios to produce zinc ferrite NPs with variable doping levels. As the temperature increases reaching the decomposition temperature of the precursors, the nucleation and growth steps of nanocrystal formation were initiated and decomposition products such as acetone, water and carbon dioxide^35^ build up and pressurize the vessel in accordance with the ideal gas law.

To facilitate Zn^2+^ doping without affecting the size and morphology of the product due to different thermodynamic and kinetic factors, the total amount of Fe(acac)_3_ and Zn(acac)_2_·*x*H_2_O was kept constant while their ratio was systematically varied as shown in Table 1 to produce zinc ferrite NPs with different compositions as confirmed by ICP-OES or UV-Vis spectroscopy (Fig. S4, ESI†). The yields of the syntheses based on cleaned product (removal of excess TREG) were calculated according to eqn (1) and were found to be 35 ± 2% for iron oxide NPs and 40 ± 3% for Zn_0.4_Fe_2.6_O_4_ NPs with no statistical difference between them.

**Table 1:** Concentration of precursors used to prepare different Zn^2+^ doped ferrites, their crystallite size *d*_XRD_ and *d*_TEM_ are given as the mean value obtained at reflection (311) with the corresponding standard deviation, lattice strain, saturation magnetisation *M*_S_ and blocking temperature *T*_B_. Values shown are averages of at least three syntheses per composition. The volume of triethylene glycol was constant at 20 ml

Fe(acac)_3_ (mM)	Zn(acac)_2_·*x*H_2_O (mM)	Obtained materials	*d*_XRD_ (nm)	*d*_TEM_ (nm)	Lattice strain (%)	*M*_S_ (emu g^−1^)	*T*_B_ (K)
200	0	Fe_*x*_O_*y*_	10.3 ± 0.7	10.1 ± 2.0	1.15 ± 0.08	80 ± 7	221 ± 23
187	13	Zn_0.2_Fe_2.8_O_4_	—	—	—	—	—
174	26	Zn_0.4_Fe_2.6_O_4_	9.9 ± 1.6	10.2 ± 2.5	1.1 ± 0.2	108 ± 3	175 ± 13
153	47	Zn_0.7_Fe_2.3_O_4_	—	—	—	—	—
133	67	ZnFe_2_O_4_	—	—	—	—	—

### Structural analysis

**Fig. 1:**
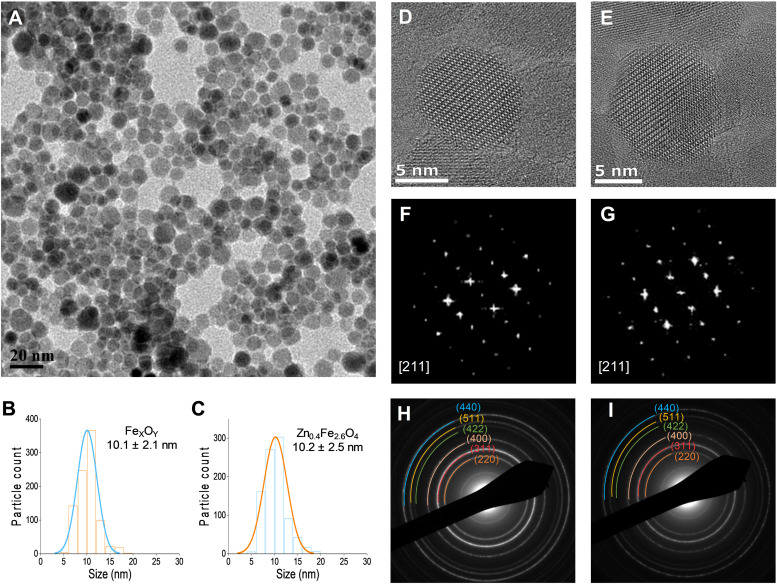
(A) TEM image of Zn_0.4_Fe_2.6_O_4_ NPs prepared under high temperature with autogenous pressure; (B) and (C) size distributions; (D) and (E) HRTEM images; (F) and (G) FFT patterns down the [211] axis; and (H) and (I) selected area electron diffraction (SAED) patterns with denoted crystal planes for iron oxide and Zn_0.4_Fe_2.6_O_4_ NPs respectively.

A typical transmission electron micrograph of the synthesized spherical Zn_0.4_Fe_2.6_O_4_ NPs is shown in Fig. 1(A). The histograms of spherical iron oxide NPs and spherical Zn_0.4_Fe_2.6_O_4_ NPs are shown in Fig. 1(B) and (C). The sizes of these NPs were obtained by measuring 300 NPs from each of three independent syntheses, then compared and showed no statistical difference (*p* > 0.05). High resolution transmission electron microscopy (HRTEM) showed a single phase throughout the NP volume in both iron oxide and Zn_0.4_Fe_2.6_O_4_ syntheses as shown in Fig. 1(D) and (E) while the Fourier transform (FFT) patterns shown in Fig. 1(F) and (G) reveal high periodicity within the NPs. Selected area electron diffraction (SAED) shows diffraction rings of spinel structures in both nanomaterials as indexed in Fig. 1(H) and (I). These results suggest that further analogous analyses between the two nanomaterials are possible since the iron oxide NPs and Zn_0.4_Fe_2.6_O_4_ NPs prepared by this high temperature with autogenous pressure method are morphologically and structurally the same.

**Fig. 2:**
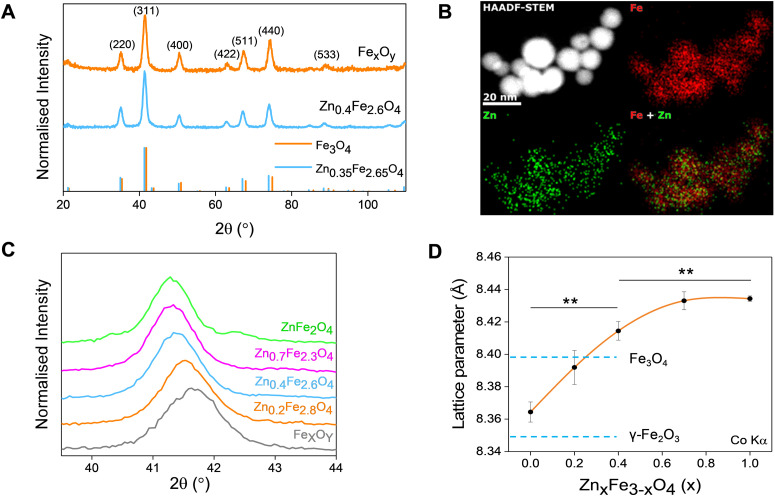
(A) XRD patterns of iron oxide and Zn_0.4_Fe_2.6_O_4_ NPs (ICDD references 01-075-0449 and 01-086-0510 for Zn_0.35_Fe_2.65_O_4_ respectively); (B) elemental mapping of iron and zinc by HAADF-STEM; (C) magnified region around the (311) XRD diffraction peak; (D) dependence of unit lattice parameter on the level of Zn^2+^ doping (*n* = 3). Values reported as mean ± SEM. *p* Values were calculated based on a two-tailed *t* test: ** indicates *p* < 0.01.

Thermal decompositions of Fe(acac)_3_ and Zn(acac)_2_.xH_2_O were carried out (Fig. S5, ESI†) and showed a significant difference of the temperature where they start decomposing (∼100 °C). Further characterization such as powder X-ray diffraction (XRD) (Fig. 2(A)) and HAADF STEM with EDX mapping (Fig. 2(B)) were performed to rule out the formation of a ZnO phase prior to the formation of ferrites leading to a possible core shell structure.

The uniform distribution of zinc and iron throughout the NP volume and in combination with SAED (Fig. 1(H) and (I)) and XRD diffraction patterns (Fig. 2(A)) show that only spinel based materials are formed. The uniform distribution of zinc and iron also excludes the formation of a Zn_*x*_Fe_3−*x*_O_4_@ZnO or Fe_*x*_O_*y*_@ZnO core–shell architectures. A magnification of the most intense peak (311) of the diffraction pattern of spinel structures is shown in Fig. 2(C) shows a shift to lower value in correlation with increasing the Zn^2+^ doping level. In accordance with a change of occupancy between the interstices of the lattice with cations of different ionic radius, the diffraction peaks are expected to move to lower values with increased doping.

The effect of Zn^2+^ doping on the lattice parameter (calculated using eqn (4) and (5)) is shown in Fig. 2(D). The lattice parameter increases with increasing Zn^2+^ doping before reaching a plateau. This is in agreement with Zn doped cobalt ferrite NPs, the lattice parameter increased with the amount of Zn^2+^ doping.^36^

In the case of maghemite (γ-Fe_2_O_3_), the structure includes vacancies in octahedral interstices 
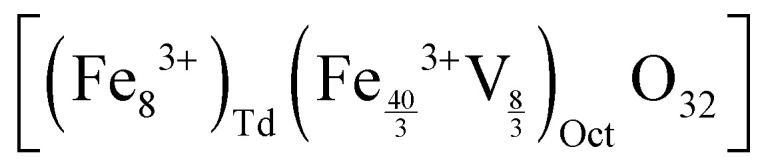
 while magnetite (Fe_3_O_4_) does not have vacancies 
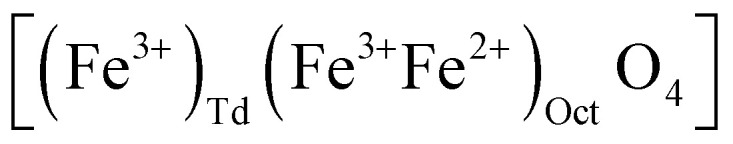
 and consequently has an increased lattice parameter^37^ (highlighted with dashed blue lines) shown in Fig. 2(D).

The introduction of Zn^2+^ displaces tetrahedral Fe^3+^ to octahedral interstices which changes the degree of inversion of the inverse spinel structure of iron oxide to a normal spinel.

The ionic radius of Zn^2+^ is bigger than the ionic radius of high spin Fe^3+^ which it displaces.^38^ Hence the lattice expansion to accommodate the cation substitution, which is reflected in the lattice parameter shown Fig. 2(D). Using Vegard's law shown in eqn (6) which describes the molar fraction of two solids in a mixture using the lattice parameter of materials, an estimate of the composition of the iron oxide NPs used herein was determined. The estimated content of Fe_3_O_4_ and γ-Fe_2_O_3_ is 50% for both making the iron oxide NPs used herein a Fe_3_O_4_@γ-Fe_2_O_3_ composite.

In addition to TEM size measurements, the crystallite size of NPs was determined from XRD patterns using the Scherrer equation shown in eqn (2) as shown Table 1 and are in good agreement with each other.

By combining the crystallite size, the peak position and broadening of the peak, a Williamson–Hall analysis, eqn (3), can yield the lattice strain of the NPs which is related to interatomic bond changes, vacancies and defects.^39^ Since the magnetic properties of these materials are tightly correlated to the interatomic coupling between cations at different interstices within the lattice structure, an increase in strain would be detrimental. As shown in Table 1 the lattice strain of the NPs does not change by the introduction of Zn^2+^ in the lattice (*n* = 3, *p* > 0.05).

### Magnetic characterization

**Fig. 3:**
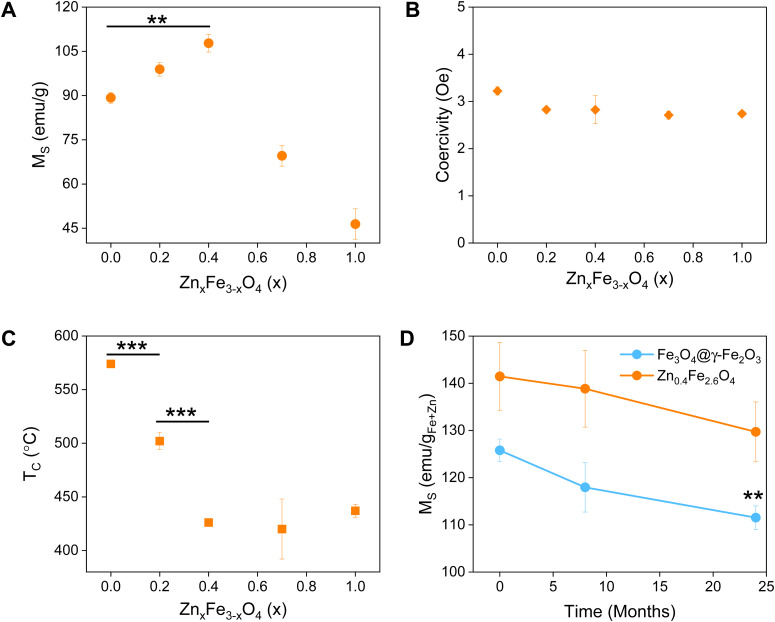
Different properties of synthesized Fe_3_O_4_@γ-Fe_2_O_3_ and ferrites with various levels of Zn^2+^ doping: (A) saturation magnetization in emu g_sample_^−1^ (*M*_S_); (B) coercive field; (C) Curie temperature (*T*_C_); and (D) long-term monitoring of the *M*_S_, values were compared between 0 and 24 month for Fe_3_O_4_@γ-Fe_2_O_3_. Values reported as mean ± SEM, *n* = 3, *p* values were calculated based on a two-tailed *t* test: *** indicates *p* < 0.001, ** indicates *p* < 0.01 and * indicates *p* < 0.05.

The synthesized NPs were characterized by means of their *M*_S_, superparamagnetism, Curie temperature (*T*_C_) and long-term magnetic stability as shown in Fig. 3.

The magnetic properties of Fe_3_O_4_ have been well described.^37^ Herein, we evidence that the introduction of Zn^2+^ cations in the crystal with a d^10^ configuration and preferential localization in the tetrahedral sites of the lattice enhances the magnetic properties of the material.^40,41^

The zinc ferrites exhibit complex doping-dependent *M*_S_ values with an observed increase of the *M*_S_ before diminishing with further increasing doping levels. An optimal doping level when the highest *M*_S_ values (108 emu g_sample_^−1^) were obtained is approximately 13.5% with a molecular formula of Zn_0.4_Fe_2.6_O_4_ which agrees with previous studies. Examples of zinc ferrite NPs published in literature with their corresponding size and *M*_S_ are tabulated in Table S1 (ESI†).

Among isotropic zinc ferrite NPs, the highest *M*_S_ (105 emu g_sample_^−1^) was obtained from 22 nm NPs (5.57 × 10^−24^ m^3^) prepared under high temperature conditions. The isotropic Zn_0.4_Fe_2.6_O_4_ NPs presented herein, prepared under high temperature with autogenous pressure have a 10.2 nm diameter (0.56 × 10^−24^ m^3^) and 110 emu g_sample_^−1^. Our NPs having 2 times smaller diameter and 10 times less volume, possess the highest *M*_S_. Equally important is the superparamagnetic nature of the NPs presented which makes them suitable for biomedical applications with low coercivity (Fig. 3(B)) and T_B_ values below room temperature (Table 1), unlike the next most magnetic zinc ferrite NPs with *T*_B_ values above ambient temperature (ref. 19, Table S1, ESI†).

A reduced effective anisotropy, *K*_eff_ value was obtained for Zn_0.4_Fe_2.6_O_4_ (1.21 × 10^−5^ J m^−3^) compared to iron oxide NPs (1.52 × 10^−5^ J m^−3^) as shown in Fig. S6 (ESI†).

The softening of the material is also seen in the T_C_ values obtained where a reduction of >150 °C was observed for Zn_0.4_Fe_2.6_O_4_ in comparison to Fe_3_O_4_@γ-Fe_2_O_3_ NPs (Fig. 3(C)).

**Fig. 4:**
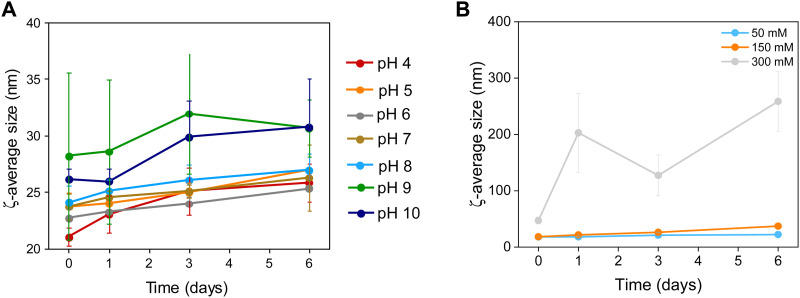
Colloidal stability of Zn_0.4_Fe_2.6_O_4_ NPs at (A) different pH values and (B) a range of electrolyte concentrations (*n* = 3).

Surface atoms could reach up to 30% for a 10 nm ferrite NP,^42^ a spin-canting effect becomes even more important and is detrimental to the *M*_S_ as illustrated in Fig. S7 (ESI†), however the Zn_0.4_Fe_2.6_O_4_ still have high *M*_S_. Over time and as Fe^2+^ oxidizes to Fe^3+^ iron oxide-based nanomaterials might lose their magnetic properties. In the case of zinc ferrites, partial replacement of Fe^2+^ with Zn^2+^ which is redox inactive at ambient conditions extends the magnetic stability of the NPs as shown in Fig. 4(D).

Given that other high temperature reactions exist in literature describing the synthesis of magnetic NPs, we investigated the possible role of pressure in producing high quality NPs.

### Effects of pressure

In order to evaluate the role of pressure in NP synthesis and how it might relate to the preparation of high-quality nanomaterials, NPs prepared at ambient pressure (Fig. S8, ESI†) were used for comparison of their structural and magnetic properties. As it can be seen from Table S2 (ESI†), the size of the NPs prepared at ambient pressure is significantly smaller (7.7 ± 1.7 nm) compared to NPs prepared under autogenous pressure (10.2 ± 2.5 nm). Interestingly, a statistically significant increase in lattice strain is observed for NPs prepared at ambient pressure (1.5 ± 0.3%) compared to (1.1 ± 0.2%) and consequently a decreased *M*_S_ value to 80 ± 7 emu g_sample_^−1^ is also observed in accordance with the lattice dependent magnetic properties of the materials explained earlier. In conclusion, pressure has beneficial effects on NP synthesis producing superior materials to high temperature only synthetic protocols, it could be due to a higher crystallinity structure.

### Colloidal stability

The NP surface was studied before and after washing and a suitable functionalization protocol was developed for the successful conjugation of citrate molecules on the surface of the NPs to produce stable dispersions (Fig. S1, ESI†). The stability of the NPs was assessed in terms of colloidal stability over a range of pH and electrolyte concentrations using DLS to measure the hydrodynamic sizes of NPs. The colloidal stability was assessed over a one-week period, over a pH range between 3–12 and electrolyte concentration range between 0–300 mM to mimic physiological conditions (pH 7.2 and 150 mM NaCl), as shown in Fig. 4.

NPs had a short-lived colloidal stability at pH values of 11 and 12 (not shown) for 2 and 1 d respectively after which the hydrodynamic size increased sharply. At pH 3 the NPs precipitated immediately and hence no measurements could be obtained. NPs were stable between pH 4–10 for the duration of the study (6 d). The NPs exhibit good stability in electrolyte concentrations up to 150 mM. At 300 mM electrolyte concentration the NPs were unstable with sharp increases in their hydrodynamic size. The results suggest that the citrate stabilized NPs are stable in physiologically mimicking conditions (pH 7.2, 150 mM NaCl).

### Optical properties

**Fig. 5:**
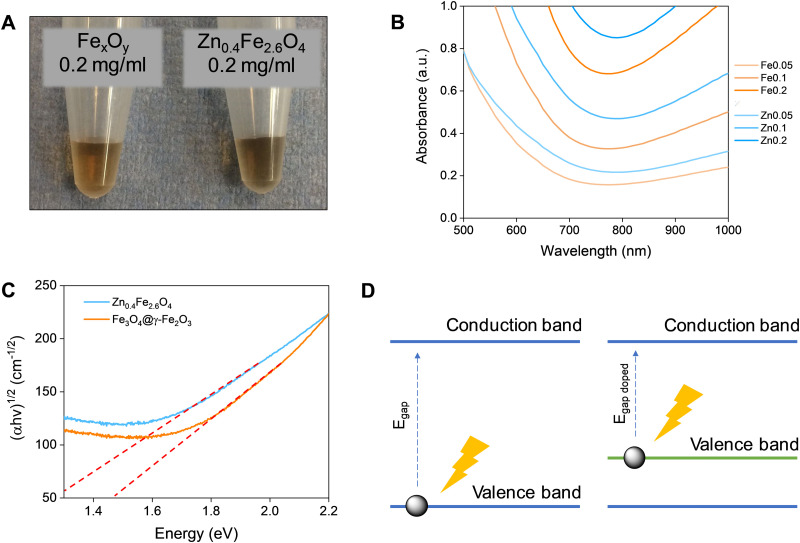
(A) Digital photographs of Fe_3_O_4_@γ-Fe_2_O_3_ (left) and Zn_0.4_Fe_2.6_O_4_ (right) NPs at a concentration of 0.2 mg_Fe+Zn_ ml^−1^. (B) UV-Vis-NIR spectra of Fe_3_O_4_@γ-Fe_2_O_3_ and Zn_0.4_Fe_2.6_O_4_ NPs for concentrations between 0.05–0.2 mg_Fe or Zn_ ml^−1^. (C) Tauc plots of Fe_3_O_4_@γ-Fe_2_O_3_ and Zn_0.4_F_e2.6_O_4_ NPs with extrapolation of the linear region to *y* = 0. (D) A scheme of bandgap decrease by doping.

Fe_3_O_4_ and Zn_0.4_Fe_2.6_O_4_ NPs have a dark colour indicating absorbance throughout the visible spectrum (Fig. 5(A)). To estimate the energy of the bandgap (*E*_gap_) of each nanomaterial, UV-Vis-NIR spectra were recorded and converted to Tauc plots shown in Fig. 5(B) and (C) respectively. The linear parts of the curves in the Tauc plots were extrapolated to *y* = 0 to yield the *E*_gap_. *Y*-Axis is a function of the absorption coefficient and the photon energy. This method reveals a 22% reduction in the *E*_gap_ of Zn_0.4_Fe_2.6_O_4_ with a value of 1.03 eV compared to 1.32 eV for Fe_3_O_4_@γ-Fe_2_O_3_ NPs illustrated in Fig. 5(D). If the mechanism of photothermal conversion is solely due to transformation of electromagnetic radiation to phonons (crystal vibrations) then Zn_0.4_Fe_2.6_O_4_ NPs should perform better in photothermia.

### Biocompatibility studies

**Fig. 6:**
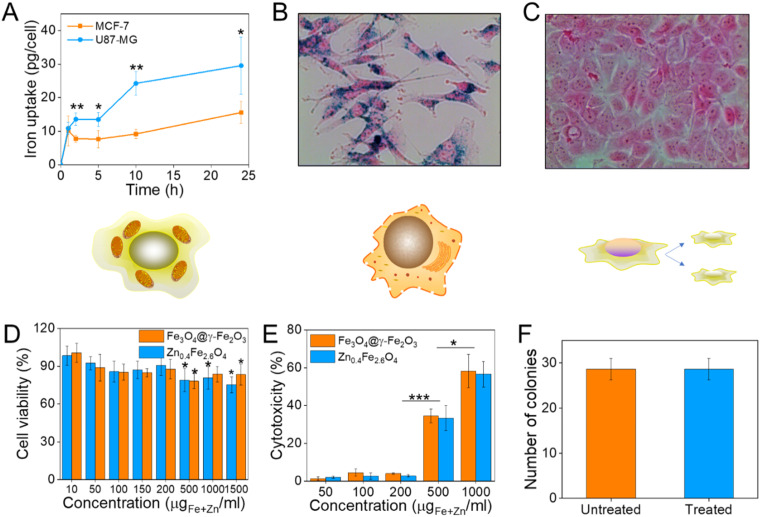
(A) Iron uptake of U87-MG and MCF-7 cells as calculated from single-cell magnetophoresis, (B) and (C) their optical microscope images when incubated with 10 μg_Fe+Zn_ ml^−1^ Zn_0.4_Fe_2.6_O_4_ NPs respectively for 24 h; (D) cell viability of U87-MG cells as measured by the MTT assay after treatment with Fe_3_O_4_@γ-Fe_2_O_3_ and Zn_0.4_Fe_2.6_O_4_ NPs at different concentrations for 24 h; (E) cytotoxicity of Fe_3_O_4_@γ-Fe_2_O_3_ and Zn_0.4_Fe_2.6_O_4_ NPs obtained by the LDH assay; (F) number of colonies formed from single-cell suspensions of U87-MG cells *via* clonogenic assay after treatment of cells with 0.2 mg_Fe+Zn_ ml^−1^ Zn_0.4_Fe_2.6_O_4_ NPs for 24 h. Values reported as mean ± SEM. *p* Values were calculated based on a two-tailed *t* test: *** indicates *p* < 0.001, ** indicates *p* < 0.01 and * indicates *p* < 0.05.

Prior to selecting a cell line to assess the cell viability after NP treatment, the NP uptake was evaluated between brain glioblastoma U87-MG and breast adenocarcinoma MCF-7 cells by single-cell magnetophoresis. U87-MG cells were uptaking the NPs more readily compared to MCF-7 as shown in Fig. 6(A) and hence were used to assess the toxicity of the materials. To visualize the uptake of NPs, both cell lines were incubated with NPs for 24 h and then the cells were washed, fixed and the iron content stained with Prussian blue. The blue colour indicative of iron ions are profound in U87-MG cells while no blue colour can be detected inside the MCF-7 cells as can be seen in Fig. 6(B) and (C) respectively.

To quantify cell viability after treatment with NPs, U87-MG cells were incubated with Fe_3_O_4_@γ-Fe_2_O_3_ and Zn_0.4_Fe_2.6_O_4_ NPs for 24 h after which the reduction of the tetrazolium salt was monitored by UV-Vis spectroscopy. Neither of the materials shows significant decrease of cell viability up to 200 μg_Fe+Zn_ ml^−1^ (Fig. 6(D)). Nanomaterials decreased cell viability to 80% for concentrations over 500 μg_Fe+Zn_ ml^−1^. In addition to MTT, the enzyme lactose dehydrogenase (LDH) was measured as an indication of cellular membrane integrity, another marker for cell health. Similarly to the MTT assay, the LDH assay reveals a similar trend with concentrations of 500 μg_Fe+Zn_ ml^−1^ and above having an increased cytotoxic effect on the cells shown in Fig. 6(E). Further to metabolism and membrane integrity of cells which are short-term markers of cell health, a clonogenic assay was used to evaluate the longer-term impact of NPs. Genetic changes, *i.e.* through reactive oxygen species, can damage DNA and produce malfunctioning proteins whose accumulation can lead to apoptosis. A clonogenic assay uses the cell cycle and proliferation of cells to assess cell health. Treating the cells with a high concentration of NPs of 200 μg_Fe+Zn_ ml^−1^ would disregard damages caused by metabolic and membrane integrity effects as seen previously with the MTT and LDH assays respectively and probe any genetic changes that would manifest themselves in longer timeframes than those used in the colourimetric assays. Cells formed the same numbers of colonies whether treated with Zn_0.4_Fe_2.6_O_4_ NPs or not indicating that they are tolerated and do not alter protein expression or arrest the cell cycle, shown in Fig. 6(F). The fact that both materials exhibit the same trend in cell viability by two different assays, the unaffected proliferation when treated with NPs as well as the fact iron oxide is an FDA approved material proves that our Zn_0.4_Fe_2.6_O_4_ NPs are biocompatible and well-tolerated materials.

### Application in hyperthermia

**Fig. 7:**
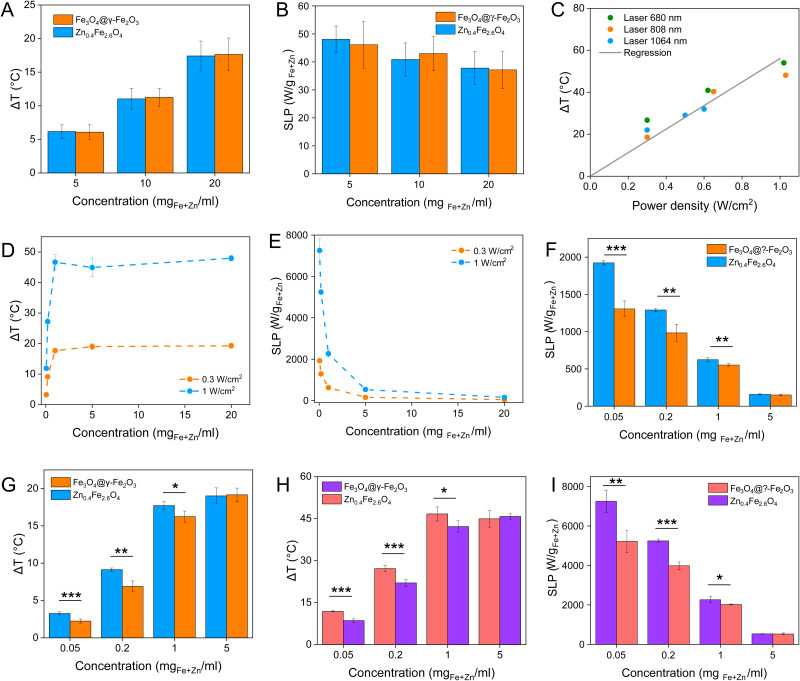
(A) and (B) are the change in temperature and SLP values respectively of Fe_3_O_4_@γ-Fe_2_O_3_ and Zn_0.4_Fe_2.6_O_4_ NPs at different concentrations under an alternating magnetic field (*f* = 342 kHz, *H* = 14.4 kA m^−1^); (C) change in temperature dependence of Zn_0.4_Fe_2.6_O_4_ NPs on the power density of different wavelength lasers at a power density of 0.3 W cm^−2^; (D) change in temperature and (E) SLP values of Zn_0.4_Fe_2.6_O_4_ NPs at different concentrations irradiated with an 808 nm laser at 0.3 and 1 W cm^−2^; (F) and (G) are the change in temperature and SLP values of Fe_3_O_4_@γ-Fe_2_O_3_ and Zn_0.4_Fe_2.6_O_4_ NPs irradiated with 0.3 W cm^−2^ at 808 nm and (H) and (I) show the same for 1 W cm^−2^. Values reported as mean ± SEM. *p* Values were calculated based on a two-tailed *t* test: *** indicates *p* < 0.001, ** indicates *p* < 0.01 and * indicates *p* < 0.05.

Magnetic NPs are typically used in MFH where their magnetic relaxation increases the internal energy of the system which dissipates to heat. Current obstacles for the clinical translation of MFH are the high concentration of NPs that need to reach the tumour due to the limited heating of superparamagnetic NPs. Other potential approaches include the preparation of anisotropic NPs which have a higher magnetothermal conversion compared to isotropic NPs. The Brezovich criterion (*H* × *f* = 5 × 10^9^ A m^−1^ s^−1^) is an upper limit of the product between the frequency and amplitude of the alternating magnetic field to prevent unspecific heating from induction of eddy currents. Herein, superparamagnetic isotropic NPs of Fe_3_O_4_@γ-Fe_2_O_3_ and Zn_0.4_Fe_2.6_O_4_ compositions were compared whilst complying with the revised Brezovich criterion. Our results indicate a magnetothermal performance typical of superparamagnetic NPs as shown in Fig. 7(A) and (B). Depending on the product of frequency and amplitude, SLP values, which is a measure of the rate of heating and absolute change in temperature vary proportionally as shown in Table S3 (ESI†). It is observed from Fig. 7(A) that the change in temperature achieved after heating the aqueous dispersions of NPs is proportional to concentration with Fe_3_O_4_@γ-Fe_2_O_3_ and Zn_0.4_Fe_2.6_O_4_ NPs producing the same amount of heat, approximately 1 °C per mg_Fe+Zn_. The SLP value is desirable to be high to prevent thermal energy from dissipating in the surrounding tissue before it can elevate the tumour temperature. This property was found to be independent of concentration of material and with a value typical of superparamagnetic NPs, in the 50–100 W g_Fe_^−1^ range (Fig. 7(B)). Changing the frequency and amplitude of the alternating magnetic field whilst remaining within the acceptable product between the two, we get the same absolute change in temperature and SLP as shown in Fig. S9A and B (ESI†). We did not observe an augmentation in performance with NPs of same size and morphology but higher *M*_S_ of Fe_3_O_4_@γ-Fe_2_O_3_ NPs vs that of Zn_0.4_Fe_2.6_O_4_ NPs according to eqn (9) and (10) showing a dependence of the power generated to the square of the *M*_S_ value of the NPs.10
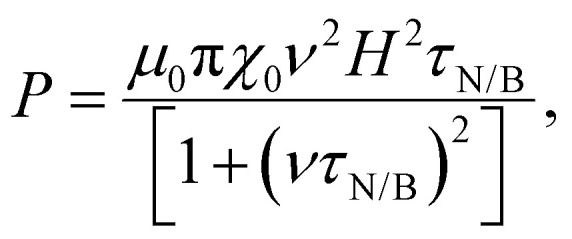
where11
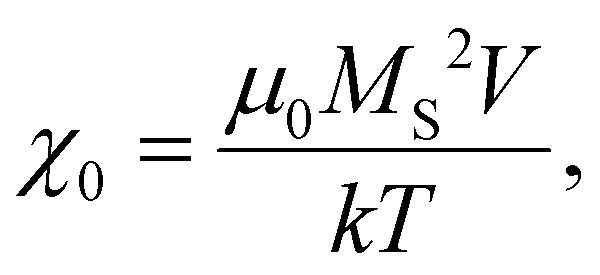
where *P* is power dissipation, *μ*_0_ is the permeability of free space, *v* is the frequency of the AC field, *H* is the magnetic field, *τ*_N/B_ is the relaxation time, *M*_S_ is the saturation magnetisation, *V* is the volume, *k* the Boltzmann constant and *T* the temperature.

From the magnetic characterization we can conclude that the introduction of Zn^2+^ causes magnetic softening and consequently a decrease in the *K* value reducing particle motion contribution to heating.

**Fig. 8:**
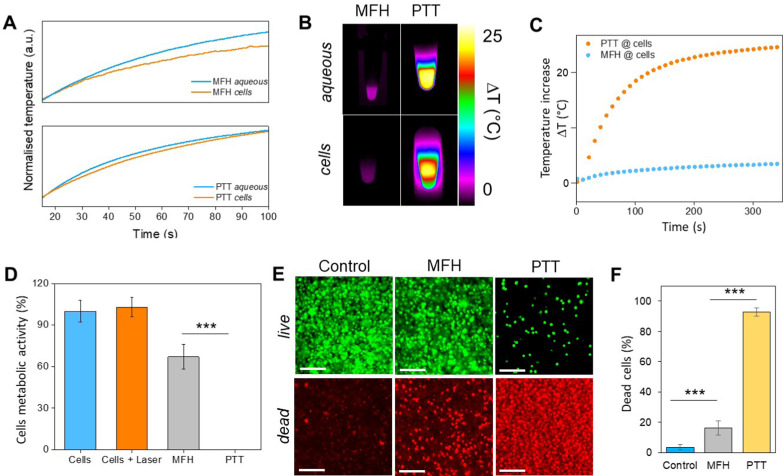
(A) Initial part of heating curves of Zn_0.4_Fe_2.6_O_4_ NPs in solution and *in vitro* (U87-MG cells) at a concentration of 2 mg_Fe+Zn_ ml^−1^ under MFH and PT treatment; (B) infrared images of Zn_0.4_Fe_2.6_O_4_ NPs in suspension and in cells at the same concentration under MFH and PT; (C) absolute temperature of U87-MG cell pellet with Zn_0.4_Fe_2.6_O_4_ NPs at 2 mg_Fe+Zn_ ml^−1^ treated with MFH and PT for 10 min each; (D) cell viability after MFH and PT treatment as obtained from the Alamar blue assay. (E) Live/Dead imaging after treatment with MFH and PT, or on untreated cells (Control). (F) Average number of dead cells (in % of total live and dead cells) over three independent experiments. Values reported as average ± SD. *p* Values were calculated based on a two-tailed *t* test: *** indicates *p* < 0.001.

Recently, the ability of iron oxide NPs to produce heat from light was evidenced.^2,7,43,44^ Herein, a thorough comparison between the two heating modalities, MFH and PT, was evaluated and for the first time, the effect of the smaller bandgap of Zn_0.4_Fe_2.6_O_4_ compared to iron oxide NPs assessed for enhanced photothermal performance. Laser wavelengths tested are within the near-infrared biological window where water and other biomolecules do not quench the light (Fig. S10, ESI†). The photothermal performance of Fe_3_O_4_@γ-Fe_2_O_3_ and Zn_0.4_Fe_2.6_O_4_ NPs is independent of laser wavelength at saturating conditions and it increases linearly with power density up to the values tested as shown in Fig. 7(C). We first calculated the light to heat conversion coefficient (see eqn (9) in the Methods section), that was found independent to the NPs concentration, and equal to 42.9 ± 5.0% for the Fe_3_O_4_@γ-Fe_2_O_3_ NPs and 43.3 ± 8.1% for the Zn_0.4_Fe_2.6_O_4_ NPs. In contrast to magnetothermal conversion, photothermal conversion saturates at very low concentrations (1 mg_Fe+Zn_ ml^−1^) with a much bigger effect (Δ*T* = 47 °C at 1 W cm^−2^ for 1 mg_Fe+Zn_ ml^−1^ using an 808 nm laser), Fig. 7(D) compared to 17 °C for MFH (20 mg_Fe+Zn_ ml^−1^), Fig. 7(A). This is explained based on the limiting factor being the incoming light and not the light absorbing material, which when enough material is present, it will absorb the whole energy of the incoming light. SLP, also a measure of heating rate, is therefore inversely proportional to concentration in photothermia (Fig. 7(E)). As the material has a dark colour with high molar attenuation coefficient (*ε* = 111.1 M^−1^ cm^−1^, derived from Fig. S11, ESI†), the higher the concentration the less penetrating distance the light can travel before it is absorbed. It is observed that Zn_0.4_Fe_2.6_O_4_ NPs exhibit a higher photothermal effect compared to Fe_3_O_4_@γ-Fe_2_O_3_ NPs in both their ability to increase to higher temperature by 5–20% and the rate (SLP) with which they do so as it can be seen in Fig. 7(F) and (G). The temperature rise was profound up to the saturating concentration of 1 mg_Fe+Zn_ ml^−1^ and increasing further with higher power density of 1 W cm^−2^ (Fig. 7(H)). The Zn_0.4_Fe_2.6_O_4_ NPs outperform Fe_3_O_4_@γ-Fe_2_O_3_ in producing higher SLP values in all concentrations (0.05–1 mg_Fe+Zn_ ml^−1^). At the minimum concentration tested of 0.05 mg_Fe+Zn_ ml^−1^, Zn_0.4_Fe_2.6_O_4_ NPs had SLP values of 1900 and 7000 W g_Fe+Zn_^−1^ for 0.3 and 1 W cm^−2^ while Fe_3_O_4_@γ-Fe_2_O_3_ had only 1300 and 5000, respectively (Fig. 7(G) and (I)). Hence a 46–40% increase in SLP was found for Zn_0.4_Fe_2.6_O_4_ NPs compared to Fe_3_O_4_@γ-Fe_2_O_3_. The higher the concentration the lower the SLP values were obtained. This is explained by the fact that light does not penetrate deep in the suspension when the concentration is high before it is absorbed and it takes longer for all the particles to absorb light. In comparison, MFH at a concentration of 1 mg_Fe+Zn_ ml^−1^ does not show any increase in temperature. Comparing the optimal conditions for obtaining high temperature increase in MFH (20 mg_Fe+Zn_ ml^−1^) and PT (1 mg_Fe+Zn_ ml^−1^), the change in temperature is the same at about 18 °C, whilst the SLP value of PT is more than 16 times higher. Repeating photothermal heating cycles of Zn_0.4_Fe_2.6_O_4_ NPs reach the same temperature indicative of their stability during treatment (Fig. S12, ESI†). The ability to increase the heating effect whilst reducing the amount of material needed might offer a solution for the clinical translation of localized hyperthermia. Both heating modalities have then been assessed in cancer cells. Previous reports showed the magnetothermal effect diminishing once the NPs are associated with the cells while the photothermal effect remains unaffected.^43–47^ This is in relation to the environment-dependent mechanism of magnetothermal conversion (reorientation in space) and the independent mechanism of photothermal conversion (production of phonons by vibrational relaxation of excited electrons). In agreement with previous studies, the NPs presented herein experienced a decrease in the magnetothermal effect (35 W g_Fe+Zn_^−1^*vs*. 45 W g_Fe+Zn_^−1^ for cellular confinement and aqueous suspension respectively), as shown in Fig. 8(A). Because these NPs have a low shape anisotropy and therefore an order of magnitude less *K*_eff_ compared to other NPs such as magnetosomes, nanocubes or nanoflowers, the decrease in their SLP is less than two-fold, while over ten-fold for the latter.^43,45^ This indicates that the NPs are relying more on Néel than Brown relaxation, the first is environment independent whilst the latter is environment-dependent due to physical reorientation of the particle, retaining their heating properties in aqueous dispersions and in cells. Similarly, PT results are in agreement with literature which states that the effect remains unaffected both in suspension and in cellular confinement shown in Fig. 8(A). It is important to note that the final temperature reached remained unchanged for both MFH and PT in aqueous dispersions and in cells as shown in Fig. 8(B), only the heating rate changed slightly for MFH. Nevertheless, the change in temperature reached after incubation of Zn_0.4_Fe_2.6_O_4_ NPs at a concentration of 0.2 mg_Fe+Zn_ ml^−1^ for 24 h with U87-MG cells (final concentration in cell pellet was 2 mg_Fe+Zn_ ml^−1^) was inadequate for therapy using MFH while PT reached thermoablation-level temperatures. The effect of both thermotherapies was evaluated on live U87-MG cells (Fig. 8D–F) with MFH achieving only 30% of reduced cell metabolic activity, or 15% of cell death while PTT achieved an impressive 100% decrease of cell metabolic activity, and 93% cell death after 10 min treatment with a low laser power density of 0.3 W cm^−2^, at 808 nm wavelength. The above findings illustrate that semiconductor PT is a more efficient thermotherapy than MFH and that Zn^2+^ doping in ferrite structures enhances the photothermal effect further.

## Conclusions

A series of Zn^2+^ substituted ferrites were prepared and characterized using a high temperature with autogenous pressure synthesis. A Zn^2+^ level dependent *M*_S_ is observed with the highest value obtained for composition Zn_0.4_Fe_2.6_O_4_. The developed synthetic procedure produced material with less strain compared to high temperature-only reactions leading to the most magnetic Zn_0.4_Fe_2.6_O_4_ isotropic NP with the smallest volume reported to date. The NPs dispersions prepared exhibit good stability colloidally for 4 years in the fridge and magnetically for at least 24 months. The Zn_0.4_Fe_2.6_O_4_ NPs exhibit the same biocompatibility profile with Fe_3_O_4_@γ-Fe_2_O_3_ NPs. The magnetothermal and photothermal effects of both Fe_3_O_4_@γ-Fe_2_O_3_ and Zn_0.4_Fe_2.6_O_4_ NPs were assessed with photothermia being much more efficient than magnetothermia in both in solution and *in vitro* conditions due to the smaller bandgap of Zn_0.4_Fe_2.6_O_4_. Complete cell death of brain glioblastoma cells could be achieved after 10 min of photothermal treatment. The biocompatible, high PTT effect could have potential for translation as new modality for cancer treatment.

## Conflicts of interest

There are no conflicts to declare.

## Supplementary Material

TB-011-D2TB01338J-s001
