# Supplementary material for: Zn doped iron oxide nanoparticles with high magnetization and photothermal efficiency for cancer treatment
Source: J Mater Chem B. 2022 Oct 12;11(4):787–801. doi: 10.1039/d2tb01338j (PMC9890495; doi:10.1039/d2tb01338j)
Supplement: TB-011-D2TB01338J-s001 [file TB-011-D2TB01338J-s001.pdf]

Electronic Supplementary Material (ESI) for Journal of Materials Chemistry B.
This journal is © The Royal Society of Chemistry 2022

Supporting Information

Zn doping of ferrite nanoparticles increases magnetization and photothermal efficiency for cancer treatment

Georgios Kasparis[a,b], Anouchka Plan Sangnier[c,d], Lilin Wang[a,b], Christoforos Efstathiou[b], Alec P. LaGrow[a,b], Andreas Sergides[a,b], Claire Wilhelm[c,] and Nguyen Thi Kim Thanh[a,b,*]*

a. Biophysics Group, Department of Physics and Astronomy, University College London, Gower street, London WC1E 6BT, UK

b. UCL Healthcare Biomagnetic and Nanomaterials Laboratories, 21 Albemarle street, London W1S 4BS, UK

c. Laboratoire PhysicoChimie Curie, Institut Curie, PSL Research University - Sorbonne Université – CNRS, 75005 Paris, France.

d. Inserm, U1148, Laboratory for Vascular Translational Science, Universite Paris 13, Sorbonne Paris Cite´, Bobigny F-93017, France..

*Email: ntk.thanh@ucl.ac.uk , claire.wilhelm@curie.fr

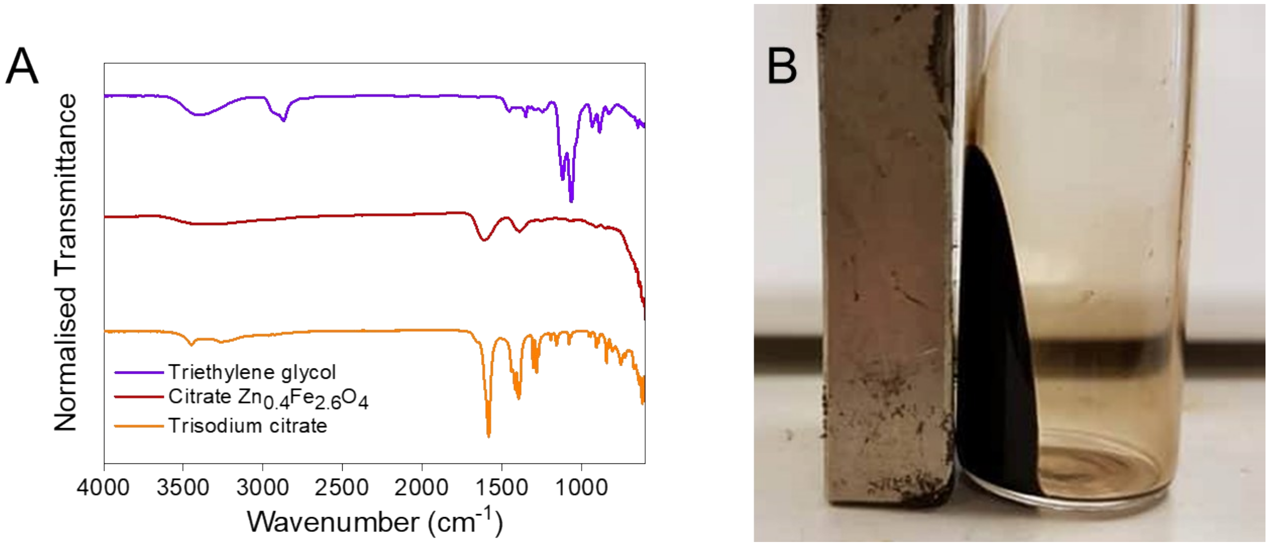

Figure S1. A) ATR-FTIR of TREG, trisodium citrate coated $Zn_{0.4}Fe_{2.6}O_4$ NPs and trisodium citrate. B) Digital image of stable trisodium citrate coated $Zn_{0.4}Fe_{2.6}O_4$ ferrofluid under the influence of a bar magnet.

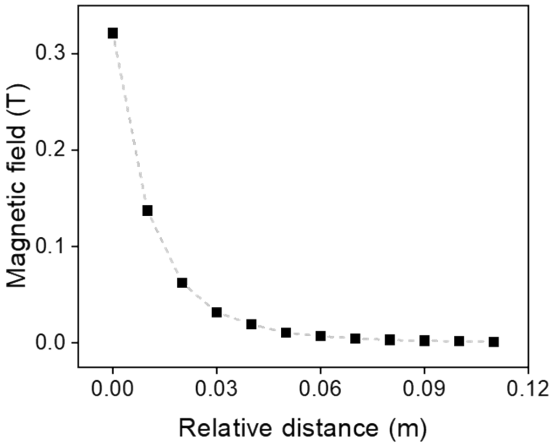

Relative Distance (m)	Magnetic Field (T)	Field Gradient (dB/dx)
0	0.321	
0.01	0.1373	-18.37
0.02	0.0626	-7.47
0.03	0.0321	-3.05
0.04	0.01945	-1.265
0.05	0.01089	-0.856
0.06	0.00717	-0.372
0.07	0.00507	-0.21
0.08	0.00337	-0.17
0.09	0.002495	-0.0875
0.1	0.001871	-0.0624
0.11	0.001535	-0.0336

Figure S2. Magnetic field measurements over distance using a gaussmeter for the bar magnet used in single cell magnetophoresis.

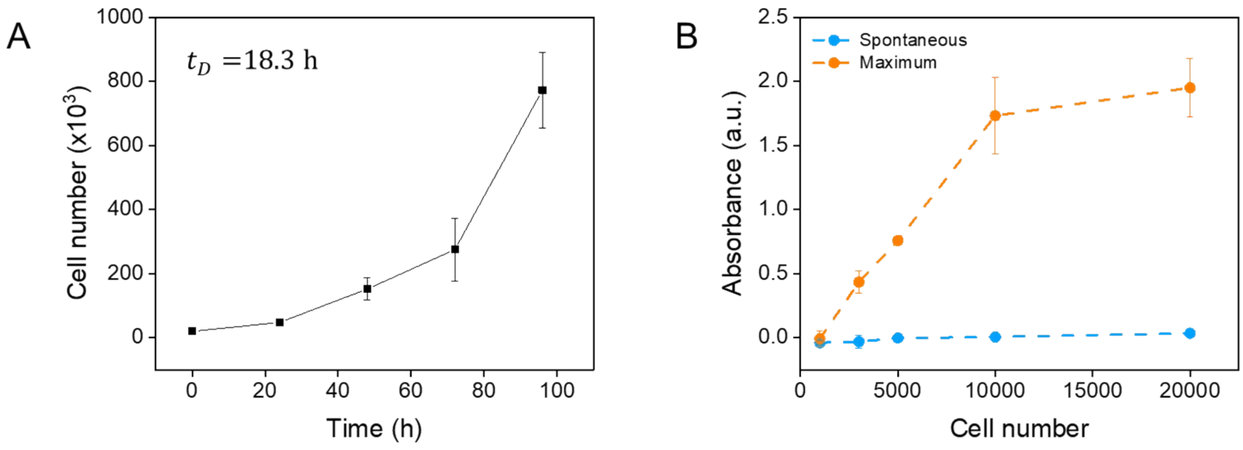

Figure S3. A) U87MG cell growth curve. B) Spontaneous and Maximum LDH activity controls with respect to cell number for optimum cell seeding number.

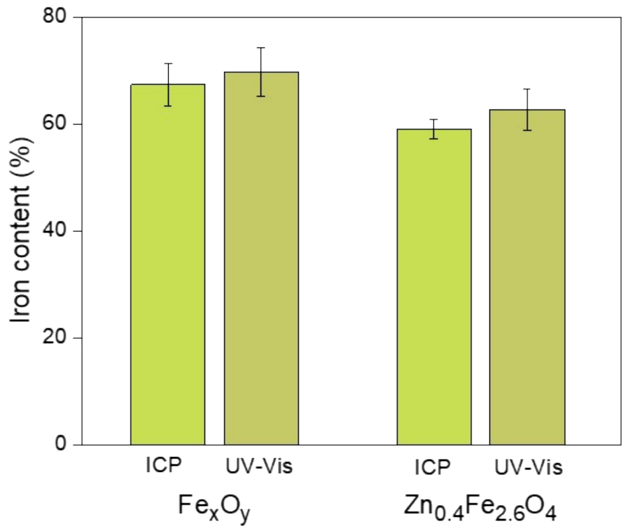

Figure S4. Comparison of iron content in Fe_3O_4@γ-Fe_2O_3 and $Zn_{0.4}Fe_{2.6}O_4$ NPs by ICP-OES and UV-Vis measurements ($n = 5, p > 0.05$).

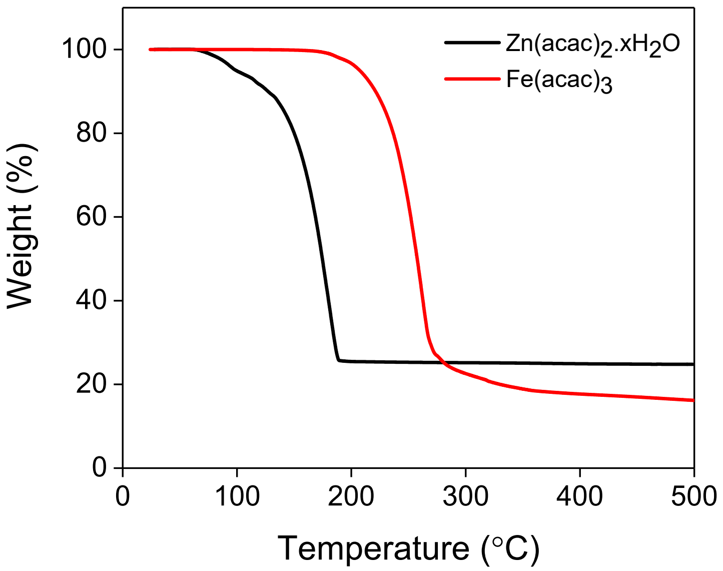

Figure S5. TGA of $Zn(acac)_2.xH_2O$ and $Fe(acac)_3$ under nitrogen.

Table S1. Zinc ferrite NPs with their respective elemental composition, average size and M_S.

Composition	Average size (nm)	M_S (emu/g)	Reference
$Zn_{0.39}Fe_{2.61}O_4$	13.4	30	27
$Zn_{0.16}Fe_{2.84}O_4$	14	97	28
$Zn_{0.4}Fe_{2.6}O_4$	15	99.3^{α}	26
$Zn_{0.34}Fe_{2.66}O_4$	17	64.6	29
$Zn_{0.4}Fe_{2.6}O_4$	22	105^{α}	19

$^{\alpha}$ value converted to emu/g of material from emu/g_{Fe+Zn}.

From T_B values the effective anisotropy (K_{eff}) can be estimated according to eq (1)

$$T_B = \frac{K_{eff} V}{k_B \ln\left(\frac{\tau_m}{\tau_0}\right)} \qquad (1)$$

Where T_B is the blocking temperature (221 K for $Fe_3O_4@\gamma\text{-}Fe_2O_3$ and 175 K for $Zn_{0.4}Fe_{2.6}O_4$ NPs), K_{eff} is the effective anisotropy, V is the volume of the NPs, k_B is the Boltzmann constant ($1.38 \times 10^{-23} J/K$), τ_m is the measurement time and τ_0 is the attempt time; the value $\ln\left(\frac{\tau_m}{\tau_0}\right)$ has the value of 25 in typical laboratory measurements:

$$T_B = \frac{K_{eff} V}{k_B \ln\left(\frac{\tau_m}{\tau_0}\right)} \quad \therefore \quad K_{eff} = \frac{T_B k_B \ln\left(\frac{\tau_m}{\tau_0}\right)}{V}$$

$$\therefore K_{eff_{Fe_3O_4@\gamma-Fe_2O_3}} = \frac{221\,K \times 1.38 \times 10^{-23}\,J/K \times 25}{0.5 \times 10^{-24}\,m^3} = 1.52 \times 10^{-5}\,J/m^3$$

$$\therefore K_{eff_{Zn_{0.4}Fe_{2.6}O_4}} = \frac{175\,K \times 1.38 \times 10^{-23}\,J/K \times 25}{0.5 \times 10^{-24}\,m^3} = 1.21 \times 10^{-5}\,J/m^3$$

$$K_{eff} = K + 6\frac{K_S}{d}$$

$$6\frac{K_S}{d}_{Fe_3O_4@\gamma-Fe_2O_3} \equiv 6\frac{K_S}{d}_{Zn_{0.4}Fe_{2.6}O_4}$$

$$\therefore K \propto K_{eff}$$

$$Since\ K_{eff_{Fe_3O_4@\gamma-Fe_2O_3}} > K_{eff_{Zn_{0.4}Fe_{2.6}O_4}}$$

$$K_{Fe_3O_4@\gamma-Fe_2O_3} > K_{Zn_{0.4}Fe_{2.6}O_4}$$

Figure S6: Calculations of the K_{eff} of Fe_3O_4@γ-Fe_2O_3 and $Zn_{0.4}Fe_{2.6}O_4$ NPs and estimation of their magnetocrystalline anisotropy (K) value.

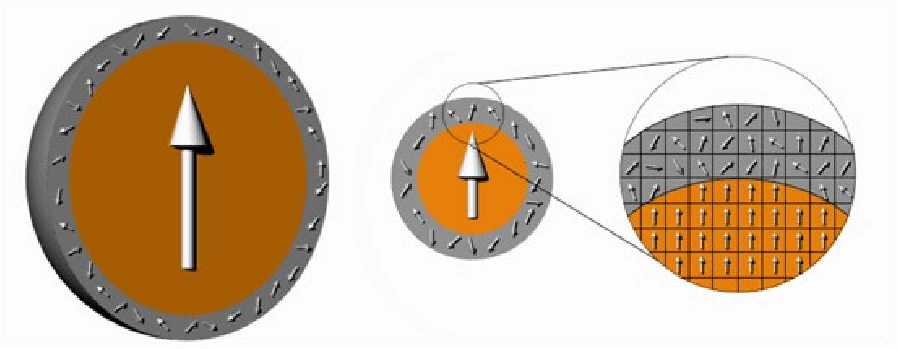

Figure S7. Illustration of the proportion of spin-canting on large versus small sized NPs.

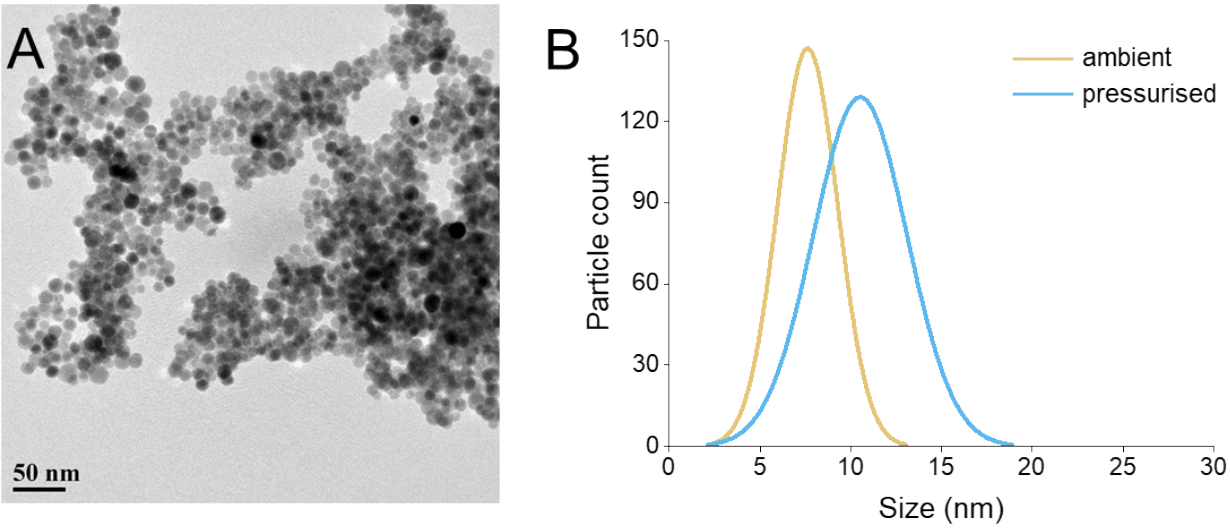

Figure S8. A) TEM of $Zn_{0.4}Fe_{2.6}O_4$ NPs prepared under ambient pressure. B) Size distribution histograms of $Zn_{0.4}Fe_{2.6}O_4$ NPs prepared at high temperature (250 °C) and ambient or autogenous pressure.

Table S2. Size, lattice strain and M_S measurements of $Zn_{0.4}Fe_{2.6}O_4$ NPs prepared under high temperature with ambient or autogenous pressure ($n = 3$). Values reported as mean ± SEM.

p-Values were calculated based on a two-tailed t-test: * indicates $p < 0.001$, ** indicates $p < 0.01$ and * indicates $p < 0.05$.**

Autogenous	10.2 ± 2.5	108 ± 3	1.1 ± 0.2
Ambient	7.7 ± 1.7	80 ± 7	1.5 ± 0.3
Significance	***	**	*

Table S3. Absolute change in temperature and SLP values of Fe_3O_4@γ-Fe_2O_3 and $Zn_{0.4}Fe_{2.6}O_4$ NPs at 20 mg_{Fe+Zn}/ml subjected to an alternating magnetic field with different product of frequency and amplitude. Values reported as mean ± SEM. p-values were calculated based on a two-tailed t-test between the change in temperature and SLP values of an alternating magnetic field with the same field amplitude but different frequency: ** indicates $p < 0.01$. n = 3.

		ΔT ± SD (°C)		SLP ± S.D (W/g_{Fe+Zn})	
Field (kA/m)	Frequency (kHz)	Fe_3O_4@γ-Fe_2O_3	$Zn_{0.4}Fe_{2.6}O_4$	Fe_3O_4@γ-Fe_2O_3	$Zn_{0.4}Fe_{2.6}O_4$
10.4	342	10.3 ± 1.2	10.7 ± 1.5	21.6 ± 2.8	23.4 ± 3.8
	471	17.9 ± 2.9	18.1 ± 2.7	38.5 ± 5.5	40.5 ± 6.8
Significance		**	**	**	**
14.4	342	17.6 ± 2.4	17.4 ± 2.2	37.2 ± 6.6	37.8 ± 5.9
	471	24.9 ± 2.2	25.0 ± 3.4	63.1 ± 6.6	63.1 ± 10.1
Significance		**	**	**	**

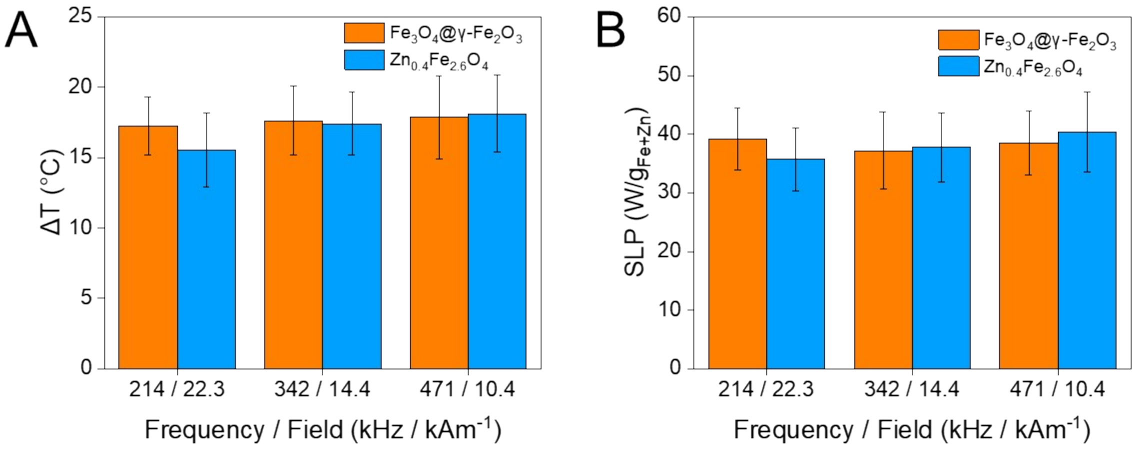

Figure S9. A) and B) is the absolute change in temperature and SLP values respectively, of Fe_3O_4@γ-Fe_2O_3 and $Zn_{0.4}Fe_{2.6}O_4$ NPs at a concentration of 20 mg_{Fe+Zn}/ml subjected to alternating magnetic fields of different combinations of frequency and field but having similar product of the two. n = 3.

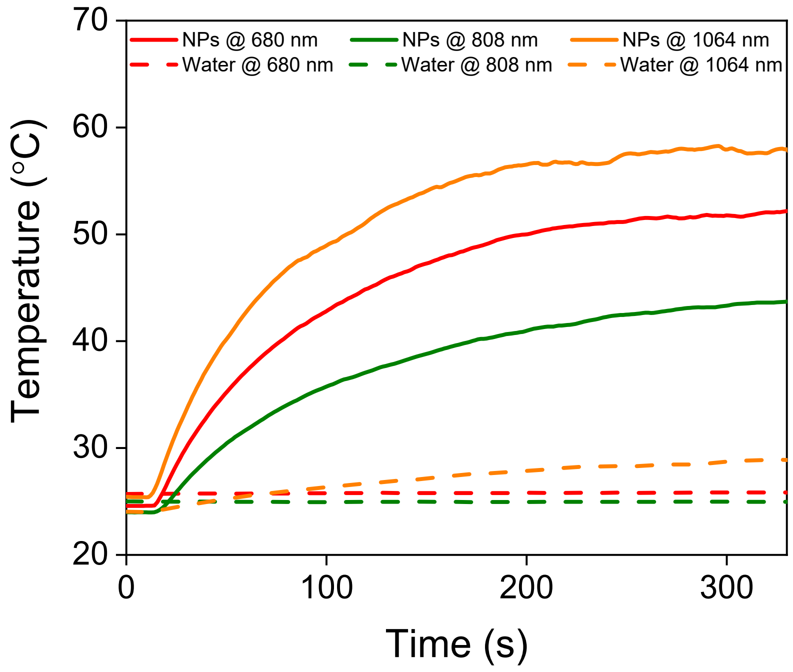

Figure S10. PT measurement of aqueous suspension of $Zn_{0.4}Fe_{2.6}O_4$ NPs and water only.

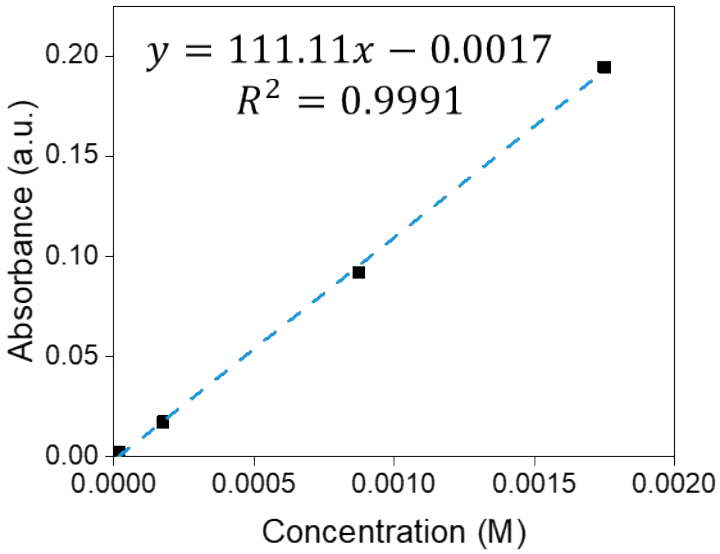

Figure S11. Absorbance of 808 nm light of $Zn_{0.4}Fe_{2.6}O_4$ NPs at different concentrations. The slope yields the molar attenuation coefficient according to Beer-Lambert law.

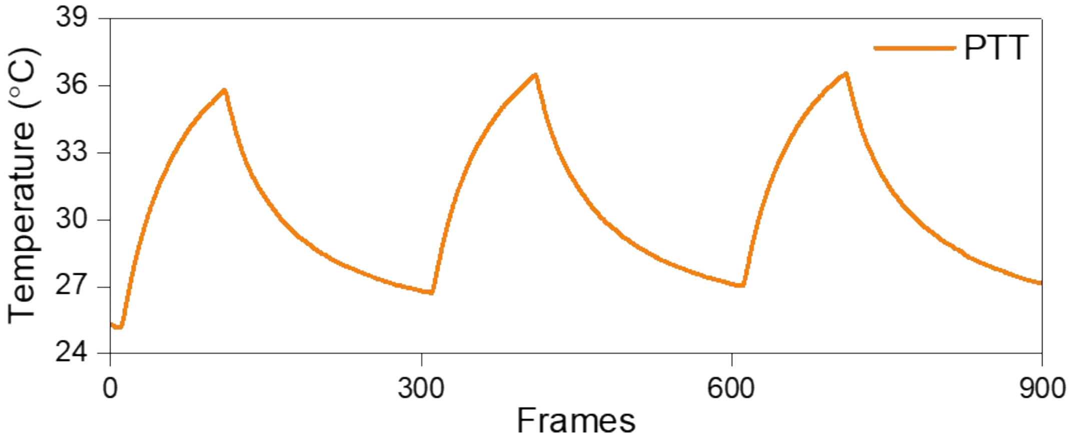

Figure S12. Photothermal heating cycles of $Zn_{0.4}Fe_{2.6}O_4$ NPs at 808 nm.
